# Reducing Falls in Older Women with Urinary Incontinence

**DOI:** 10.20900/agmr20230011

**Published:** 2024-01-25

**Authors:** Simone Reaves, Lily A. Arya, Diane K. Newman, Jean Wyman, Heather Klusaritz, Wendy Walsh, Rebecca T. Brown, Uduak U. Andy

**Affiliations:** 1 Department of Obstetrics and Gynecology, University of Pennsylvania, Philadelphia, PA, USA; 2 Department of Surgery, University of Pennsylvania, Philadelphia, PA, USA; 3 School of Nursing, University of Minnesota, Minneapolis, MN, USA; 4 Department of Family Medicine and Community Health, University of Pennsylvania, Philadelphia, PA, USA; 5 Department of Occupational Therapy, Saint Joseph’s University, Philadelphia, PA, USA; 6 Division of Geriatric Medicine, University of Pennsylvania, Philadelphia, PA, USA

**Keywords:** urinary incontinence, falls, exercise, bladder training

## Abstract

Urinary incontinence is common in older women and doubles the risk of falls in this population. The association between urinary incontinence, especially urgency urinary incontinence, and falls is multifactorial and likely the result of a complex interaction between physical, mental, social, and environmental factors. As a result of this multifactorial etiology and based on existing evidence, the integration of different fall prevention strategies including strength and resistance exercises, bladder training, and home hazard reduction have the potential to decrease the risk of falls in older women with urinary incontinence. Given the prevalence of urinary incontinence and the significant morbidity associated with falls, effective interventions to reduce fall risk in older women with urinary incontinence is of high public health significance.

## INTRODUCTION

Falls in older women have a profound impact on quality of life, and a fall may have devastating consequences. Falls and urinary incontinence are commonly co-occurring geriatric syndromes that are associated with increased morbidity [1]. Interventions to reduce fall risk in older women with urinary incontinence would address these highly prevalent conditions and would be of high public health significance.

## CO-OCCURRENCE OF URINARY INCONTINENCE AND FALLS IN OLDER WOMEN

Nearly a third of adult women over the age of 65 in the United States have experienced a fall within the preceding 12 months [2] and the rate of falls is higher in older women than men (30.3% vs 26.5%, *p* < 0.01) [2]. Women are also at higher risk of osteoporosis and reduced muscle strength, which makes their bones more likely to break during a fall [2,3]. Consequently, older women are more likely to sustain a fall-related injury (12.6% vs 8.3%, *p* < 0.01) and account for three-quarters of all fall-related hip fractures [2,3].

According to National Health and Nutrition Examination Survey (NHANES) data from 2015–2018, over 60% of community-dwelling adult women have urinary incontinence [4]. Prevalence increases with age, with over 80% of women over age 65 having any type of incontinence [4]. The most common type of incontinence in older women is urge urinary incontinence (UUI), a condition in which leakage is associated with an unexpected strong urge to urinate. Another type of incontinence is stress incontinence, or involuntary leakage of urine with activities that cause increased intra-abdominal pressure such as coughing, laughing or sneezing. Stress incontinence has not been associated with an increased risk of falls in older women [5,6], however, the risk of falls doubles in older women with UUI (OR 1.94, 95% CI 1.33–2.84) and the 12-month incidence of falls in women with even one UUI episode per week is as high as 54% [5,7]. Thus, falls in older women with UUI is a highly prevalent problem.

## ASSESSMENT AND QUANTIFICATION OF FALL RISK AND URINARY INCONTINENCE IN OLDER WOMEN

Guidelines from the American Geriatrics Society and British Geriatrics Society recommend a multifactorial falls risk assessment in older adults who have a fall and/or report gait or balance difficulties [8]. In a clinical setting, patients can be screened for falls by inquiring about a recent fall in the past 12 months or asking about difficulties with walking or balance. Gait and balance can be assessed using instruments such as the Get Up and Go Test [9], Timed Up and Go Test [10], the Berg Balance Scale [11], and the Performance-Oriented Mobility Assessment [8,12,13]. Physical function can be assessed using the Short Physical Performance Battery test (SPPB) which involves assessment of domains of balance, gait, and strength [14]. Low scores on this test have been previously reported to correspond to a high degree of mobility disability and increased fall risk [14–16]. Falls risk can also be assessed using the Activities Balance Specific (ABC) Scale, which is a validated and reliable 16-item questionnaire that asks participants to rate their confidence levels when asked to complete various physical activities in situation-specific scenarios [17].

A diagnosis of urinary incontinence can be made by asking a patient if they have had any involuntary leakage of urine in the past 12 months. The type of incontinence (stress or urge) can be determined by asking if the leakage is associated with a strong urge to urinate (consistent with urge urinary incontinence) or with an activity such as coughing, laughing or exercise (consistent with stress urinary incontinence). Women who have both stress and urge incontinence can be classified as having mixed urinary incontinence [18]. The severity of incontinence can be measured using validated instruments such as the Sandvik Severity Index, which measures frequency and quantity of urinary leakage [19].

Urinary symptoms can also be assessed using Lower Urinary Dysfunction Network (LURN) Symptom Index-29 (LURN SI-29) tool, which assesses the nature, severity and bother from a broad range of lower urinary tract symptoms [20]. The instrument was created by the NIH-NIDDK sponsored Lower Urinary Tract Dysfunction Research Network and allows for an assessment of symptoms beyond urinary incontinence, and has been shown to have internal consistency and correlates with other measures of lower urinary tract symptoms [20]. The effect of urinary incontinence on quality of life can be assessed with a tool such as the International Consultation on Incontinence Questionnaire-Urinary Incontinence Short Form (ICIQ-UI SF), which is a brief 4-item self-reported instrument to measure severity and type of urinary incontinence and its impact on quality of life [21,22]. The ICIQ-UI SF has received Grade A rating from the International Continence Society for validity, reliability, and responsiveness. Scores (range 0 to 21) are highly correlated with the pad test (*R* = 0.68) and the Incontinence Severity Index (*R* = 0.62) in women [23,24]. This instrument has been used extensively in epidemiological studies, as well as in intervention trials of various treatments for urinary incontinence [25,26].

## ASSOCIATION BETWEEN URINARY INCONTINENCE AND FALLS IN OLDER WOMEN

**Figure 1.**
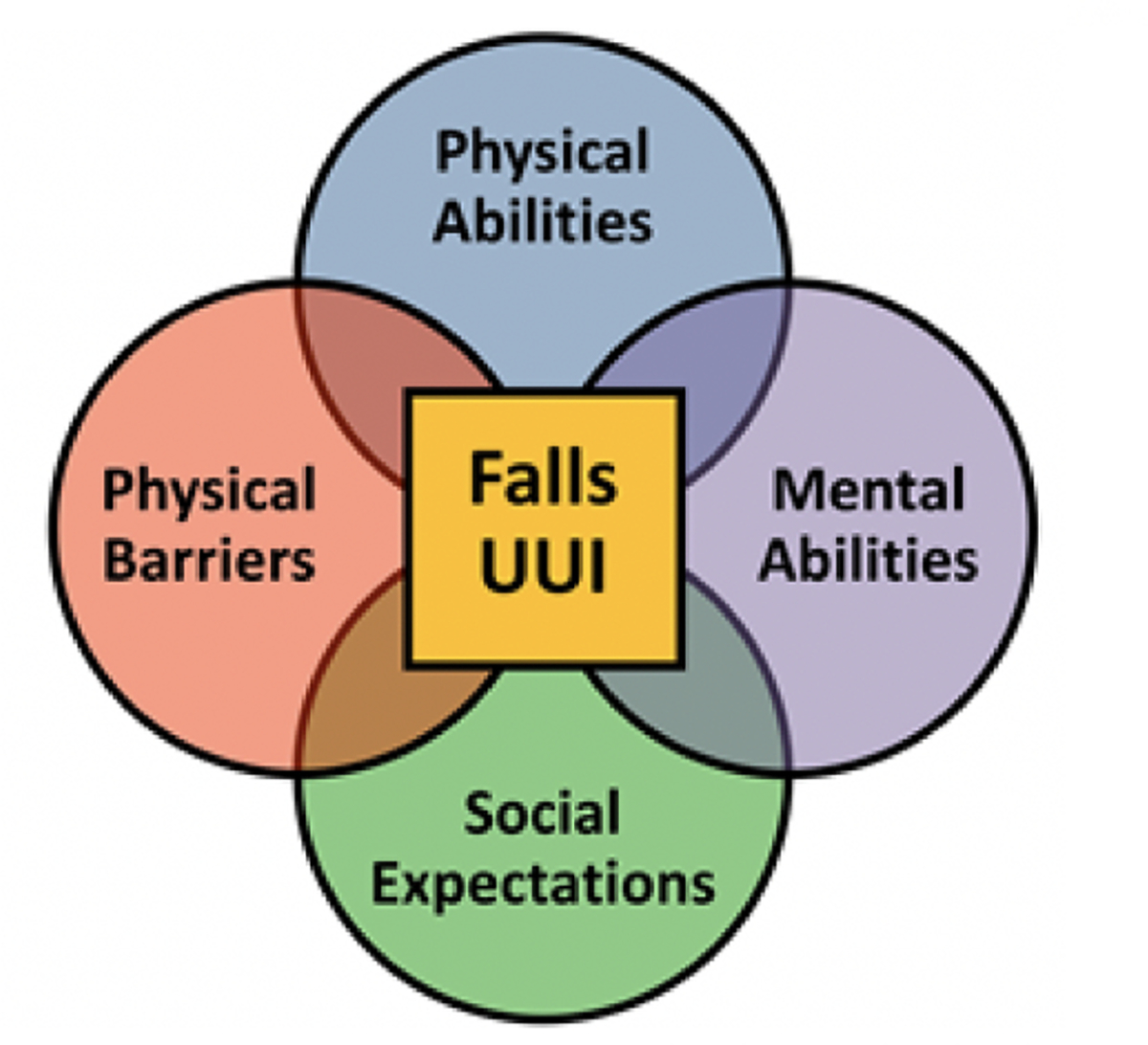
Falls in older women with UUI.

There is an association between urinary incontinence, especially urge urinary incontinence, and falls, and the reasons for this association are likely multifactorial [5,6,27,28]. A simple model in which an older woman with incontinence slips in urine and falls is not supported by evidence [29,30]. A more likely situation is one in which an older woman wakes at night with a strong urge to urinate, is anxious about leakage en route to the toilet, and may have environmental hazards in her home such as loose rugs, stairs, or absence of hand rails—all factors that increase her risk for falling. The Health Integration Theory, which posits that although the foci of illness and injury are within the body and mind, the physical and social environments contain elements that can cause or exacerbate disease and lead to injuries and disabilities [31]. Considering this framework, we have developed a bio-psycho-ecological model (Figure 1) in which falls in women with UUI are the result of a complex interaction between physical, mental, social, and environmental factors [32]. In addition to physical weakness, older women may have anxiety associated with making it to the restroom, societal roles or occupations that limit bathroom access, or environmental safety hazards that increase the risk of falls [6]. Nocturia, or waking one or more times to void during the night, is common in women and another factor that may also contribute to increased fall risk [5,33,34]. A study of 838 older women found that 85% of women older than 65 had at least one episode of nocturia and nearly 70% had two episodes. Additionally, having two or more episodes of nocturia was significantly associated with recurrent falls in this study population (OR = 1.6, 95% CI: 1.0–2.7) [35].

Urinary symptoms also closely interact with mental factors to cause falls. Women with UUI have significantly higher rates of anxiety and depression compared to continent women [36], and a strong association exists between urinary incontinence and cognitive decline [37]. Older adults with cognitive impairment are also at risk for falls due to challenges with dual-tasking while walking [38]. There is emerging evidence that urinary urgency affects gait in older adults by diverting attention from the task of walking. A small study of older adults with overactive bladder (*n* = 25) found that urinary urgency resulted in gait changes similar to those induced by a distracting task, and such changes increase risk for fall [39]. Additionally, anxiety and depression are also worse in women with UUI who have already suffered a fall. In a cross-sectional study of over 5000 adults, age 70 or older, participants with both incontinence and falls were significantly more likely to report symptoms of feeling upset or distress and perception of worse quality of life than those with incontinence alone [7]. It is therefore critical that an effective falls intervention program address urgency-related anxiety.

Decline in physical performance has been associated with urinary incontinence in older women [40–42]. Existing literature supports a bidirectional relationship between physical function and urge urinary incontinence: worsening urinary incontinence is associated with worsening physical function including increased fall risk, and conversely, deterioration of physical function is associated with the development of urinary incontinence [7,41–46]. This decline in physical performance may also impact quality of life. In a small study of women over age 70 with symptomatic urinary incontinence, functional status was evaluated using the Modified Physical Performance Test (MPPT) and Short Physical Performance Battery (SPPB), as well as physical performance measures such as Timed Up and Go (TUG). There was an association between lower MPPT score and worse TUG performance with greater UI impact on quality of life [47]. Furthermore, sarcopenia, defined as a decrease in skeletal muscle mass and/or strength, has also been associated with urinary incontinence in older women [44]. Therefore, optimizing physical function and strength has potential for reducing urinary incontinence, and inversely, treating urinary incontinence may improve physical performance and strength and prevent falls in high-risk older women.

## FALL PREVENTION IN OLDER WOMEN WITH URINARY INCONTINENCE

According to the Center for Disease Control (CDC), unintentional falls are the top cause of injury-related morbidity and mortality in the United States [48], and therefore, fall prevention in older adults is of great public health interest. Based on existing evidence, the United States Preventative Services Task Force (USPSTF) has supported the implementation of exercise and multifactorial interventions for fall prevention in older adults [49], however, data is lacking on the impact of these established fall prevention strategies as well as novel approaches specifically focused on women with urinary incontinence.

### Strength and Balance Exercise

Fall prevention exercise programs, especially those that incorporate balance and functional exercises with resistance training, reduce the risks of falls in community dwelling adults. Several studies have examined the efficacy of exercise interventions on fall prevention. A large systematic review of randomized controlled trials of exercise programs (mostly multicomponent exercise incorporating aerobic, strength and balance training) in adults over age 60 found that exercise significantly decreased fall risk (*n* = 20 RCTs; 4420 participants; RR, 0.88; 95% CI, 0.79–0.98) and injuries associated with falls (9 RCTs; 4481 participants; RR, 0.74; 95% CI, 0.62–0.88) [50]. Additionally, a recent systematic review examining exercise interventions for fall prevention among older community-dwelling adults found that when compared to control, exercise reduced the rate of falls by 23% [51]. There is also evidence that interventions that included any type of exercise alone, as well as programs that combined exercise with various other strategies, can both be effective strategies to decrease injurious falls in older adults [52]. This growing body of evidence supports the benefits of exercise on reducing fall risk in older women; however, whether these strategies can be applied to women with urinary incontinence still needs to be established. Barriers to exercise also must be considered; for some women with urinary incontinence, urinary leakage may pose a barrier [53]. In fact, a focus group of older women identified urinary leakage and shame associated with leakage as a barrier to participating in exercise [54]. Women may make adaptive behavioral changes to avoid or decrease leakage during exercise, such as voiding prior to activity, taking breaks to void, restricting fluid intake, or avoiding specific exercises altogether [55].

### Treatment of Incontinence

There is a paucity of literature regarding the effect of treatment of urinary symptoms on the risk of falls in older women with urinary incontinence. Pelvic floor muscle training (PFMT) is a recommended first line conservative management of urinary incontinence in women [18], but there is not strong evidence that PFMT alone reduces fall risk. However, when PFMT is combined with other functional exercises, there may be benefit in decreasing fall risk. A study of 88 older women who underwent PFMT combined with cognitive and balance strategies showed improvement on the performance-oriented mobility assessment (POMA) and a 21% reduction in fall risk [56]. Additionally, an increase in physical activity has been associated with a reduced risk of urinary incontinence [57], and there is emerging evidence that combining physical activity (such as walking and strength training) with behavioral treatment can benefit frail women with urinary incontinence [58].

Medical treatment of UUI can include use of medications also used for overactive bladder (OAB), such as anticholinergics or beta agonists [18]. There is evidence that medical treatment of OAB treatment reduces fall risk. A study of older adults with OAB found that medical treatment of OAB was associated with a lower rate of falls (OR = 0.88; 95% CI 0.80–0.98) [59]. Additionally, it has been shown that reducing UUI in older women by a combination of behavioral measures and medication improves self-efficacy for physical activity, which is a necessary first step for participating in an exercise program [60]. These findings are supported by those of Klay and Marfyak who demonstrated in a small cohort of 27 older women in a long-term care facility that a tailored program of anticholinergic medication and behavioral interventions reduced the number of falls by >50% in the year after the intervention [61]. These findings demonstrate the importance of incorporating medical treatment of urinary symptoms in a falls-prevention intervention for older women with UUI.

However, one must also consider the growing body of evidence concerning adverse effects associated with use of anticholinergic medications in older adults. Several studies have reported that treatment with anticholinergic medication is associated with an increased risk of cognitive decline and falls [62–64]. Furthermore, a study of patients presenting to the emergency department found that patients taking an anticholinergic medication for OAB were twice as likely to present to the emergency department for a fall (OR = 2.34) [65]. Considering these risks, anticholinergic medications should be used with caution in older women with UUI, and other medications such as beta agonists should be used if possible.

### Home Hazard Reduction

Environmental hazards are implicated in as many as one-third of all falls among older adults [66]. Common home hazards that may increase fall risk include loose rugs, electrical cords, unsteady furniture, poor lighting, and lack of stair banisters or bathroom grab rails [66]. A Cochrane review found that that home safety assessment and modification interventions were effective in reducing fall risk (RR 0.88, 95% CI 0.80 to 0.96) and were more effective when delivered by an occupational therapist and as part of a multicomponent intervention for fall reduction [67]. The American Geriatrics Society (AGS) recommends environmental assessment and modification by a healthcare professional for older adults at risk for falls [8].

### Integrated Programs

Based on the available data, strength and balance exercise, treatment of incontinence, and home hazard reduction may have a role in decreasing fall in risk in older women with urinary incontinence. Combining one or more of these approaches may have a multiplicative impact on reducing fall risk in this vulnerable population. The multifactorial etiology for the association between fall risk and UI further supports the importance of an intervention that addresses the different components. Some prior work has examined the impact of these integrated (or multicomponent) interventions on fall risk in women with UI.

A 2011 randomized study by Kim et al. examined the impact of integrated physical exercise and incontinence treatment using PFMT in among 61 women (mean age, 79 years) with multiple geriatric syndromes such as functional decline, urinary incontinence, and fear of falling [68]. Participants were randomized to an exercise plus PFMT group (*n* = 31) or a control group that received health education (*n* = 30). In the exercise group, there was a statistically significant decrease in functional decline from 50% of participants to 16.7%. The intervention group also had statistically significant improvement in urinary incontinence, from 66.7% at baseline to 23.3% after the initial intervention and 40% at 6-month follow-up. The control group showed no significant improvement [68]. Talley et al. conducted a pilot study of 42 frail older women living in independent and assisted living apartments (mean age, 84.9 years) comparing behavioral treatment of UI and exercise (*n* = 23) with a no-treatment control group (*n* = 19). The intervention group participated in 150 min of walking per week and twice weekly strength training. Over 81% of the treatment group reported improvement in incontinence, compared to 36% of the control group (χ^2^ = 4.84, *P* = 0.01) [58]. In a 2007 study by Kim et al. of 70 community-dwelling older women (mean age, 76.6 years) randomly assigned to an exercise intervention group (*n* = 35) or control group (*n* = 35), the exercise group had significant improvements in maximum walking speed (*p* = 0.04) and muscle strength (*p* < 0.001) [69]. Over half of the intervention group (54.5%) had resolution of urinary incontinence versus only 9.4% of the control group (*p* < 0.001) [69]. Schnelle et al. completed a randomized controlled trial of 190 nursing home residents (85% female, mean age 88 years) comparing a low-intensity functional exercise intervention (*n* = 92) versus a control group (*n* = 98) [70]. Compared to the control group, there was a lower rate of falls in the intervention group (OR 0.46, *p* < 0.04). The intervention group also had improved strength and mobility as well as improvement of urinary and fecal incontinence [70].

There is increasing evidence that home-based programs can be effective in fall prevention [67]. Clemson et al. examined the efficacy of a home-based intervention that integrated principles of balance and strength training into daily routines for adults ages 70 and older with a fall history [71]. Compared to a control group, participants in the integrated program saw a 31% reduction in the rate of falls [71]. Nelson et al. identified that a home-based progressive exercise program in older adults improved functional performance compared to a control group that received an education intervention [72]. Additionally, previous studies have found that a home-based exercise program delivered via DVD resulted in clinically significant improvement in physical performance in older adults [73].

Taking a home-based exercise intervention one step further by incorporating bladder training coupled with a home hazard assessment has enormous potential to benefit women with urinary incontinence. Our team has developed and tested a multicomponent intervention called ExerciseUP (Exercise and Urge Suppression Program) that addresses the factors in our conceptual model (Figure 1) and is specifically tailored for older women with UUI. Exercise UP includes: 1) strength and balance exercises 2) behavioral bladder training with urge suppression techniques and 3) home hazard assessments. In a pilot randomized controlled trial of ExerciseUP versus usual care in older women with UUI, we established the feasibility and acceptability of the intervention with high adherence rates [32]. We noted a significantly greater improvement in urinary incontinence severity scores in the intervention group [32]. Importantly, although we were not powered to assess falls and we had a short intervention duration of 6 weeks, we noted a greater improvement in falls risk score in the intervention group, although not statistically significant. In this pilot study, 68% of participants had at least one home hazard present at baseline, further underscoring the importance of the home hazard assessment as part of a multicomponent intervention [32]. Given these promising results, we have now embarked on a larger appropriately powered randomized clinical trial to assess the efficacy of the ExerciseUP intervention.

## CONCLUSIONS

Falls and urinary incontinence are commonly co-occurring geriatric syndromes that increase morbidity for older women. Strength and balance exercise interventions have been studied as an effective means to reduce fall risk and, when combined with bladder training interventions that improve urinary incontinence and home hazard assessments that improve the safety of the home, may further decrease the risk of falls in older women with urinary incontinence. Home-based interventions are a promising way to implement exercise programs in this highly vulnerable population. A home-based integrated exercise and bladder training program has immense potential to simultaneously address two prevalent geriatric syndromes and could greatly decrease morbidity for older women. Further investigation is needed to evaluate the efficacy of integrated programs as well as identify barriers and facilitators of implementation.

## Data Availability

No data were generated from the study.
